# Discovery of a polystyrene binding peptide isolated from phage display library and its application in peptide immobilization

**DOI:** 10.1038/s41598-017-02891-x

**Published:** 2017-06-01

**Authors:** Xu Qiang, Keyong Sun, Lijun Xing, Yifeng Xu, Hong Wang, Zhengpin Zhou, Juan Zhang, Fang Zhang, Bilgen Caliskan, Min Wang, Zheng Qiu

**Affiliations:** 1 School of Life Science and Technology, China Pharmaceutical University, Nanjing, 210009 P.R. China; 2 Jiangsu Collaborative Innovation Center of Chinese Medicinal Resources Industrialization, School of Pharmacy, Nanjing University of Chinese Medicine, Nanjing, 210023 P.R. China

## Abstract

Phage peptide display is a powerful technique for discovery of various target-specific ligands. However, target-unrelated peptides can often be obtained and cause ambiguous results. Peptide PB-TUP has been isolated repeatedly in our laboratory on different targets and we conducted a research on PB-TUP phage to investigate their binding properties and rate of propagation. ELISA and phage recovery assay demonstrated that PB-TUP phage had a significant superior affinity to polystyrene solid surface compared with control phage clones. In this study, some incidental bindings are excluded like blocking agents and non-specific binding of secondary antibodies. Propagation rate assays of the selected phage clones showed that the growth rate of PB-TUP phage was not superior to the control phages. Furthermore, the binding of PB-TUB to polystyrene was concentration dependent and varied with solution pH. Molecular modeling revealed that stable structures of α-helix and β-turn may contribute to the binding of PB-TUP to polystyrene plate. The PB-TUP sequence was fused to the N-terminus of peptide P2 and the fusion peptide significantly increased the binding affinity to polystyrene. The fusion peptide also enhanced the cell adhesion ability of peptide P2 with human umbilical vein endothelial cell (HUVEC). The addition of the polystyrene binding peptide provided a convenient method for peptide immobilization.

## Introduction

Phage display is an important technique for discovery of target-specific ligands for proteins or other given targets. Due to its economical, rapid, and effective properties, it has been widely used in basic research, therapeutics, diagnostics, vaccine development and epitope mapping since established by George P. Smith in 1985^1^. Antibodies, proteins and peptides can be displayed on phage surface and used for target screening. Peptides are small, poorly immunogenic and often able to penetrate through cellular membranes, therefore they offer clear advantages in comparison with other displayed molecules. The most commonly used peptide display library is Ph.D. peptide library series (New England BioLabs, Inc., USA), in which peptides are fused to the minor coat proteins (pIII). Numerous researches have been conducted with Ph.D. libraries. In another approach, phage library with major coat proteins (pVIII) bearing peptides can also be used for target screening. For instance, landscape phage library with random octapeptides have been proved an ideal resource of high-throughput screening for specific targets by Liu’s group^2–6^.

However, this technique also propounds some binding opportunities for non-target components such as polymer materials^7^, streptavidin^8^ and protein A^9^, Fc regions of the antibody^10^, biotin^11^, and blocking agents^12, 13^. In addition, phage clones that have propagation advantage could result in the enrichment and affect accuracy of phage display bio-panning results. Those peptides that do not have real affinity to targets are called target-unrelated peptides (TUPs)^12, 14^. A series of confirmed TUPs have been found in many bio-panning experiments and caused the enrichment of false-positive clones. It is necessary to discriminate whether the positive clones screened from the phage display library are TUP sequences.

In our laboratory, the Ph.D.-12 phage display peptide library was used to find the binding peptides of different target proteins. A same VHWDFRQWWQPS-displaying phage (PB-TUP) was isolated on the purpose of different targets screening and it was proved that it had binding ability to three targets by phage ELISA. This gave suspicions that it might be a target-unrelated peptide. By searching this peptide sequence, we found it was not previously identified as an unrelated peptide, but was reported by several groups as the target binding sequence^15–17^. We investigated the binding and propagation properties of this phage clone and proved that the displayed peptide was a polystyrene binding peptide. Such polystyrene binding sequence (PS-tag) could be used for improving polystyrene plate binding of peptides^18–21^, allowing site specific immobilization of proteins^21^ or antibodies^22^ directly onto the polystyrene plates with minimal conformational change. In previous work, we developed a peptide P2 which was functional in tumor growth inhibition^23^ and protection from acute inflammation^24^. We tried to improve the surface binding ability of P2 by connection of the PB-TUP peptide to the N-terminus of P2. The fused peptide showed a significantly increased binding affinity to polystyrene comparing with native peptide P2 and its activity to mediate HUVEC adhesion was maintained and improved. PB-TUP has the potential application in peptide immobilization.

## Results

### Phage binding to polystyrene with different washing buffers and blocking agents

**Figure 1:**
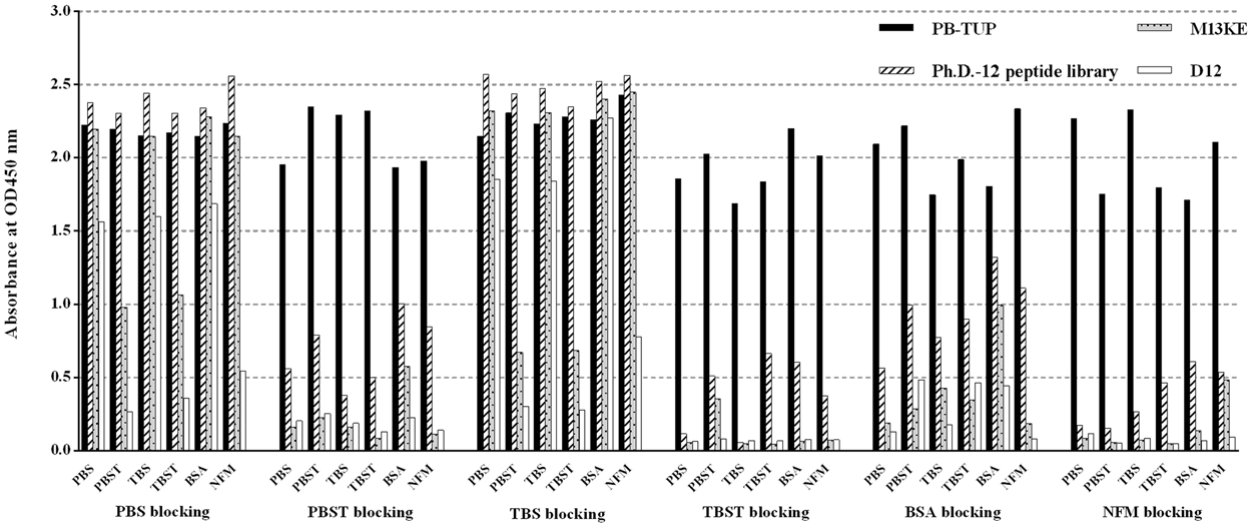
Polystyrene binding of PB-TUP, Ph.D.-12 peptide library, M13KE, and a non-relevant phage clone (D12) determined by ELISA. Four phage clones were added to the microtiter plates and the wells were treated with six different blocking buffers (first line) and six different washing buffers (second line), separately. ELISA values were used to evaluate the binding ability of these four phage clones to polystyrene. NFM had the best blocking effect. PBST and TBST were more effective washing buffers than the others. PB-TUP phage clone always had much higher absorbance comparing with the other phage clones. The experiments were performed in triplicates and repeated twice.

The binding affinities to polystyrene of phage clone PB-TUP, M13KE, Ph.D.-12 peptide library and a non-relevant control (D12) were evaluated under the treatments of different blocking agents and washing buffers. As described in the materials and methods section, blocking buffers include PBS, 0.05% PBST, TBS, 0.05% TBST, 3% BSA and 3% NFM. As shown in Fig. 1, when PBS and TBS were used as blocking buffers, absorbance of different phage clones could not be distinguished and false positive results could not be excluded. 3% NFM-TBS was the most efficient blocking reagents for avoiding nonspecific bindings of clones to polystyrene wells. Notably, PBST and TBST were more effective washing buffers comparing with the other agents. Tween 20 in the buffer plays a role in removing phage clones from nonspecific adsorption due to weak binding by hydrophobic force. Based on the results, 3% NFM-TBS was chosen as a blocking agent and 0.05% TBST was used as the washing buffer to investigate whether the PB-TUP phage clone could specifically bind to the polystyrene in the following experiments. From Fig. 1, we can see that phage clone PB-TUP always had significant higher absorbance comparing with M13KE, Ph.D.-12 peptide library and D12 under different treatment conditions. We used the same amount of phages in different groups and the capsid proteins were the same among different phage clones. Therefore, it was the displayed peptide that resulted in different affinities to polystyrene. On the basis of these findings, we concluded that polystyrene binding of PB-TUP phage clone might be due to the peptide sequence rather than phage capsid protein.

### Evaluation of phage binding to blocking buffer

**Figure 2:**
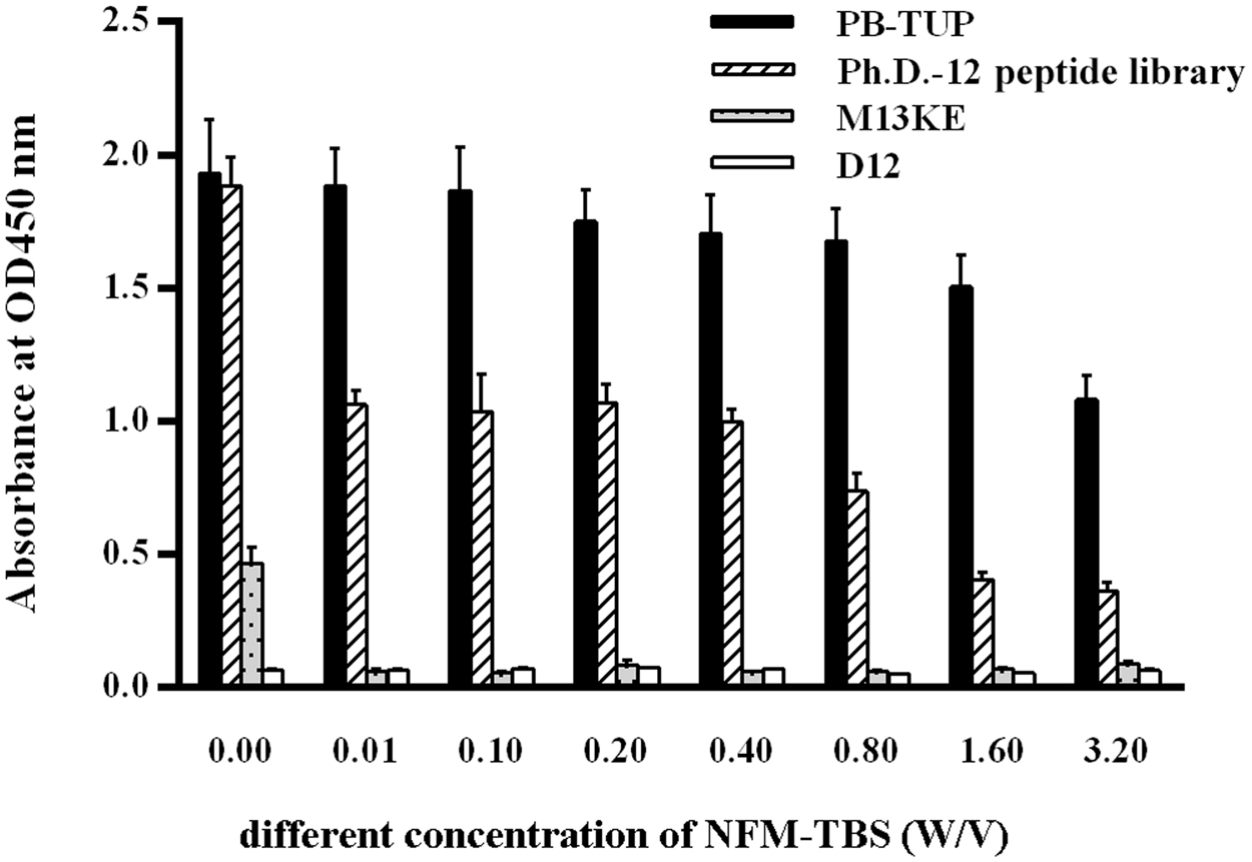
Evaluation of the phage binding to polystyrene by ELISA with different concentration of NFM-TBS as the blocking buffer. TBS with 0.00, 0.01, 0.10, 0.20, 0.40, 0.80, 1.60, 3.20% (w/v) NFM was used as blocking buffer to inhibit the adsorption of the phage clones to polystyrene. Higher concentration of NFM-TBS was needed to decrease polystyrene binding of PB- TUP phage comparing with the other phage clones.

In our study, NFM-TBS was used as the blocking buffer to prevent non-specific binding of phages. However, proteins in milk might adsorb on the surface of polystyrene and become potential binding targets for phages. In this step, we searched the possibility of binding of PB-TUP with blocking buffer components rather than with polystyrene. In order to evaluate the blocking effect of NFM-TBS, we used increasing concentrations of NFM in TBS as the blocking reagent. NFM-TBS inhibited the adsorption of all phage clones (PB-TUP, Ph.D.-12 peptide library, M13KE or D12) to polystyrene in a concentration-dependent manner, and PB-TUP phage had a higher binding than the other clones (Fig. 2). It showed that a higher concentration of NFM-TBS was necessary to reduce the plastic binding of PB-TUP comparing with the binding of M13KE, Ph.D.-12 peptide library and D12 phages. When we increased NMF concentration, binding of PB-TUP to plate was decreased and it was demonstrated that PB-TUP phage clone bound directly to polystyrene with high affinity rather than binding to blocking solution of NFM-TBS.

### Evaluation of antibody binding to polystyrene

**Figure 3:**
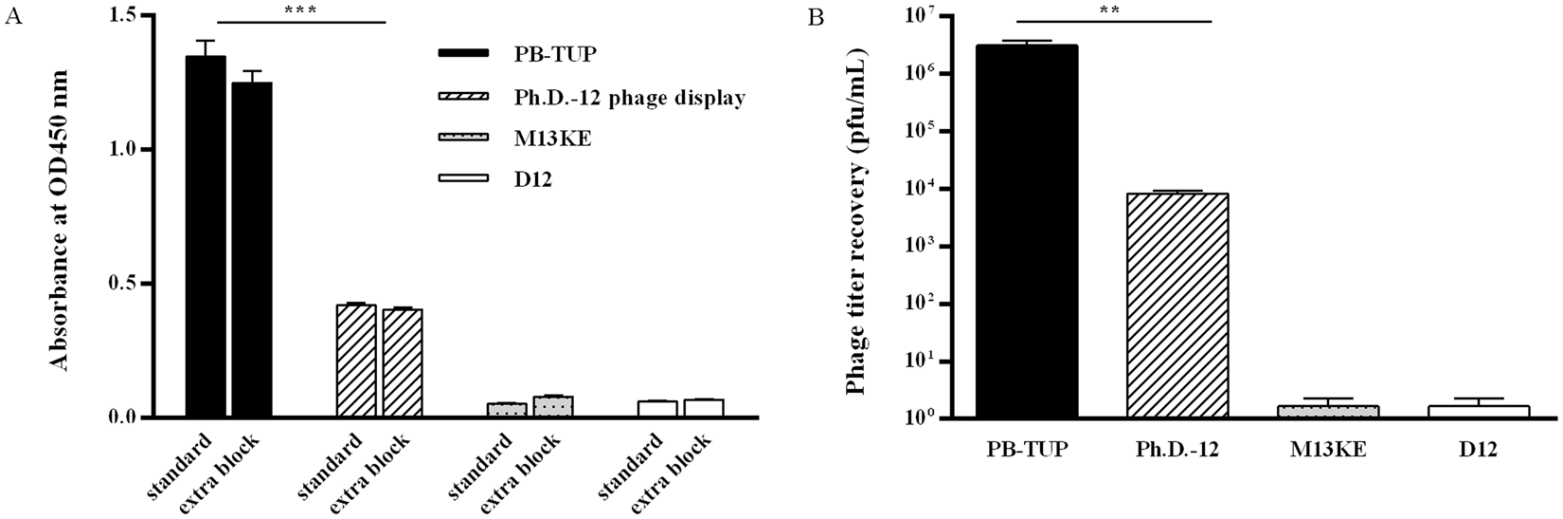
Evaluation of secondary antibody binding to polystyrene. (**A**) The standard protocol lack of extra NFM-TBS blocking versus extra blocking before adding HRP-conjugated anti-M13 antibody in ELISA. No significant differences were found between these two ELISA conditions of PB-TUP binding. Significant differences in binding ability were observed between PB-TUP clone and other clones. (**B**) 10^10^ Phage clones were added to the microtiter plates separately. Polystyrene binding phages were recovered by acid elution and titrated. There were significant differences between the phage titer of recovered PB-TUP clone and other phage clones.

HRP conjugated anti-M13 antibody is used to detect the existence of the binding phage, which is a critical step in the phage ELISA analysis. It is thought that antibody adsorption to polystyrene may cause false positive results because of non-specific bindings to antibodies and the most possible reason for antibody adsorption to polystyrene surface is incomplete blocking or excessive washing. To exclude the nonspecific binding of the secondary antibody, the microtiter wells were blocked with NFM-TBS for another 1 hour before adding HRP-conjugated anti-M13 antibody and we did not encounter a significant difference comparing with the normal procedure without extra blocking (Fig. 3A). At the same time, polystyrene-bound phages were eluted and phage titer was determined. We analyzed the phage titer recovery numbers of PB-TUP phage, M13KE, Ph.D.-12 peptide library and the D12 phage. According to the results, it was proved that PB-TUP phage had significantly high affinity to polystyrene (Fig. 3B). With these experiments, possible non-specific binding of secondary antibody was also excluded.

### Phage propagation rate

**Figure 4:**
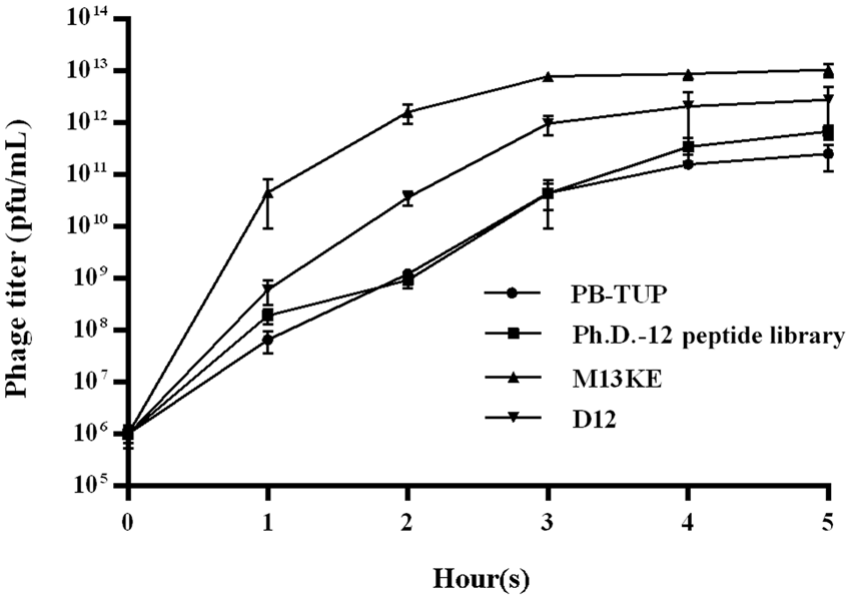
Propagation rate of phage clones. 10^6^ each of the four phage clones were individually added into the logarithmic growth phase culture of ER2738 for infection and amplification for 5 hours at 37 °C. Titer of each clone was quantified every 1 hour by counting plaques on LB/IPTG/X-gal plates. PB-TUP did not show propagation advantage compared with the other phage clones. M13KE phages had a faster growth rate than the other clones.

Enrichment of phage is not only due to specific binding to a target protein or a solid phase surface, but is also due to propagation advantage. Mutant clones with a faster propagation rate might be selected as false positive clones after several rounds of panning. To exclude this possibility, we compared the propagation rate of PB-TUP phage with the Ph.D.-12 peptide library, M13KE and D12 phages. As shown in Fig. 4, there was no statistically significant difference between the propagation rate of PB-TUP phages and the Ph.D.-12 peptide library or the D12 phages, while M13KE phages had a growth advantage over the other clones because of the fewer burdens it carries^25–27^. This experiment excluded the effect of propagation rate and proved the specific binding of the PB-TUP phage to polystyrene.

### Phage binding in a concentration dependent manner

**Figure 5:**
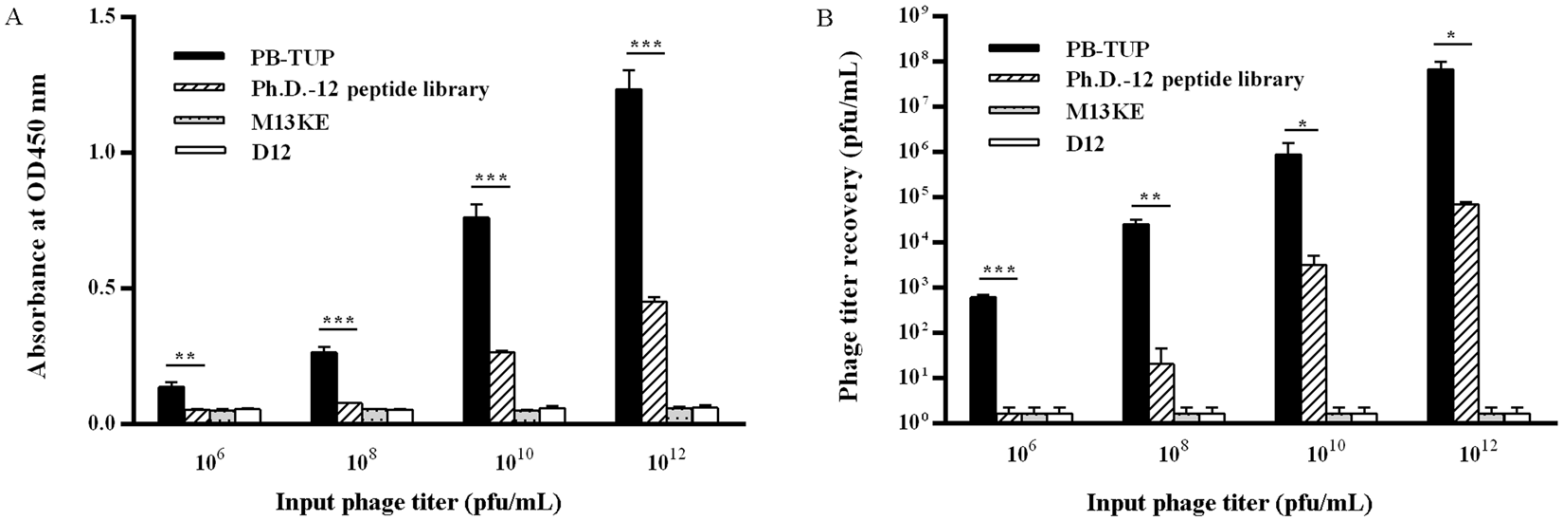
Concentration dependent binding to polystyrene. Different amounts (10^6^, 10^8^, 10^10^ and 10^12^) of the phages were added to the microtiter wells and the binding phages were measured by the absorbance (**A**) or titers of recovered phages (**B**). All phage clones bound to polystyrene in a concentration dependent manner. PB-TUP phage exhibited a significantly stronger binding comparing with the other groups in both ELISA absorbance and phage recovery.

To investigate the relationship between a phage concentration and its adsorption, various concentrations of phage (10^6^, 10^8^, 10^10^ or 10^12^) were applied to the blocked wells. ELISA absorbance and phage recovery ratio were measured. It was exhibited that PB-TUP phage was capable of performing significant polystyrene binding with increased concentrations comparing with other groups in ELISA (Fig. 5A). Phage recovery ratio also showed a similar result (Fig. 5B). This further demonstrated that the specific binding of PB-TUP phage clone to polystyrene was in a concentration-dependent manner.

### Effect of pH changes on phage binding

**Figure 6:**
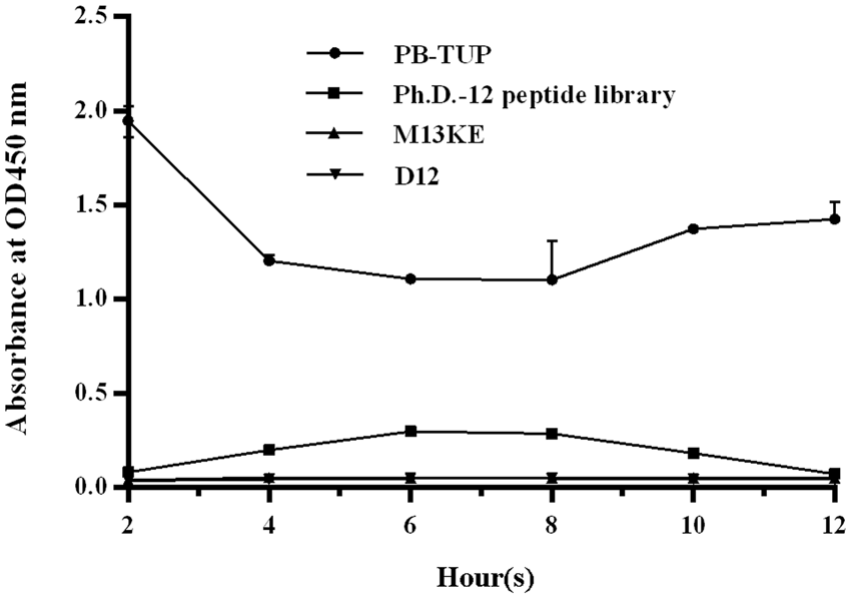
Effects of pH on phage binding to polystyrene. The binding ability of phages was analyzed under different pH conditions in ELISA. pH affected the polystyrene binding capacity of PB-TUP phage clone. Little difference was found for the other groups. Acidic and basic pH increased the binding ability of PB-TUP.

To study the effect of pH on phage binding to polystyrene, we tested the phage binding under different pH conditions from pH 2 to pH 12. The results showed that pH alterations did not affect the polystyrene binding of Ph.D.-12 peptide library, M13KE and D12 phages. However, the binding ability of PB-TUP was affected by pH change. Serendipitously, we found that binding of PB-TUP was significantly increased under acidic pH conditions comparing with the neutral pH. The binding of PB-TUP to polystyrene was increased to a less extend in basic pH (Fig. 6). The influence of pH in the binding ability might attribute to the charged amino acids of histidine, arginine and aspartic acid in PB-TUP sequence.

### Structural analysis of peptides

**Figure 7:**
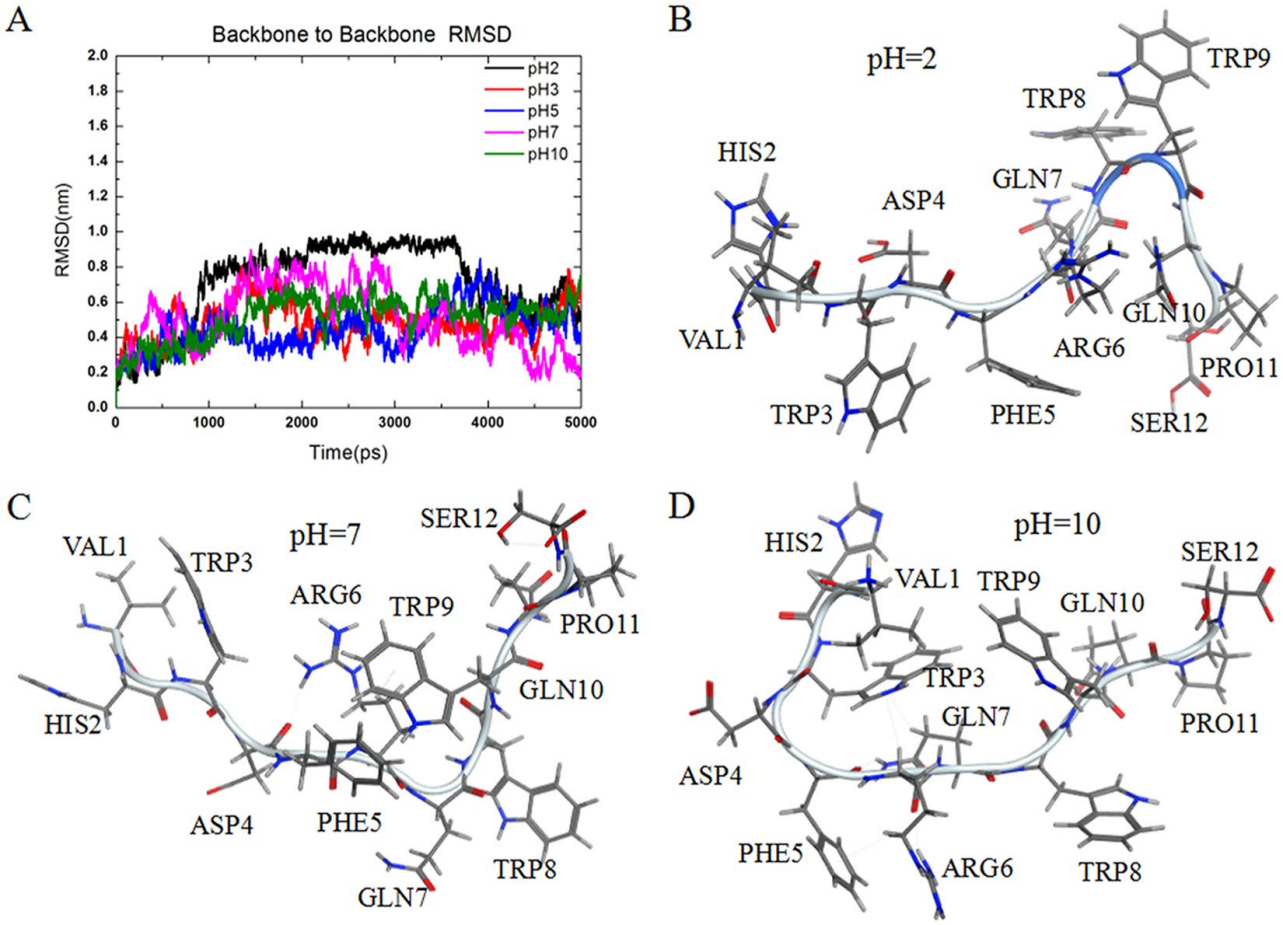
Structure of PB-TUP generated by molecular modelling. (**A**) The RMSD of all simulated structures at different pH. (**B**–**D**) Possible peptide conformations in solutions of different pH values.

Peptide secondary structures are key determinants of their interactions with other molecules. The initial structure of PB-TUP was built using ChemDraw, and then stimulated by molecular dynamics (MD) using Gromacs 5.0.2. It was demonstrated that in the simulations which were performed at 300 K temperature, all molecules displayed stable dynamics within 5 ns (Fig. 7A). It was observed that the peptide can fold up to different conformations dynamically at different pH. When pH was 2 or 10, it was also noted that the peptide can form a stable structure of α-helix (Fig. 7B) and β-turn (Fig. 7D), respectively. However, when pH was 7, the peptide adopted a random coil structure (Fig. 7C). This stable secondary structure highlighted the point of stronger binding force between peptide and polystyrene at acidic or basic pH than neutral pH.

### Binding property of P2 and PB-TUP-P2 on polystyrene surface

**Figure 8:**
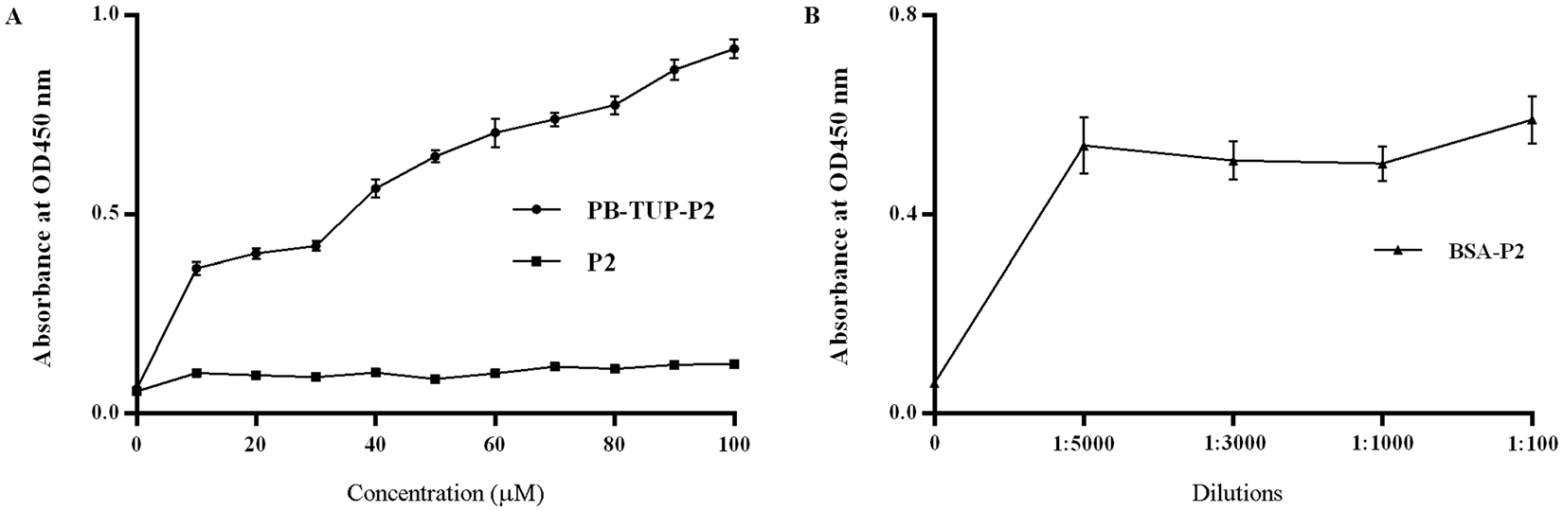
Detection of native peptide, PB-TUP fused P2 and BSA conjugated P2 by ELISA. (**A**) Binding property of P2 and PB-TUP-P2 was studied by direct ELISA using these two peptides as capture agents. P2 absorbed weakly to polystyrene surfaces, while the fused peptide PB-TUP-P2 showed a strong binding ability to polystyrene in a concentration dependent manner. (**B**) BSA conjugated P2 was used as coating agent and P2 specific antibody was used to investigate the binding of BSA-P2. We used the dilution from 1:100 to 1:5000 and not any difference was found from the ELISA value.

We generated a fusion peptide by connection of PB-TUP to the N-terminus of P2, which was developed in our group in previous study, and set up an ELISA assay to evaluate the binding ability of fused peptide. The amino sequence of peptide P2 and PB-TUP-P2 were listed in Table 1. Meanwhile, BSA conjugated P2 was studied. As a short peptide, P2 showed a poor absorption to polystyrene surface of the plate, while the fused peptide PB-TUP-P2 showed a significantly increased binding comparing with the native peptide P2. And PB-TUP-P2 showed a typical concentration dependent binding curve in Fig. 8. BSA-P2 also displayed a good absorption, although different dilutions from 1:100–1:5000 did not show any difference in ELISA value. This experiment revealed that peptide PB-TUP retained its binding ability after removal from the phage protein framework. It also proved that this peptide sequence was a potential PS-tag candidate for peptide or protein immobilization.

**Table 1:** Peptide sequence of P2 and the appended sequence with an N-terminal PB-TUP sequence.

Peptide	Sequence
P2	Pro-{D-3-Pal}-{D-Cys}-{Bip}-Arg-Gly-Glu-Gly-Gly-Gly-Gly-Ile-Val-Arg-Arg-Ala-Asp-Arg-Ala-Ala-Val-Pro
PB-TUP	Val-His-Trp-Asp-Phe-Arg-Gln-Trp-Trp-Gln-Pro-Ser
PB-TUP-P2	Val-His-Trp-Asp-Phe-Arg-Gln-Trp-Trp-Gln-Pro-Ser-{Acp}-Pro-{D-3-Pal}-{D-Cys}-{Bip}-Arg-Gly-Glu-Gly-Gly-Gly-Gly-Ile-Val-Arg-Arg-Ala-Asp-Arg-Ala-Ala-Val-Pro

### HUVEC adhesion

**Figure 9:**
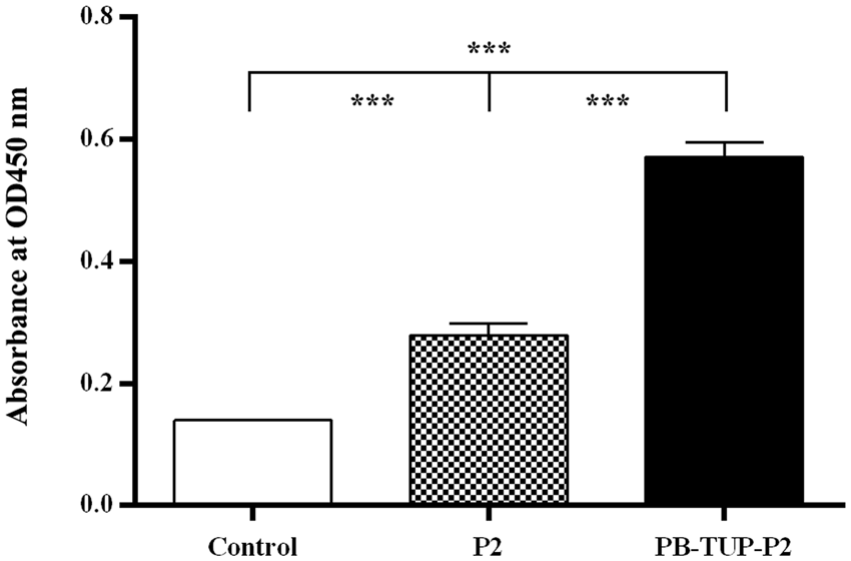
HUVECs adhesion assay of P2 and PB-TUP-P2. Polystyrene 96-well cell culture plates were coated with P2 or PB-TUP-P2 at a concentration of 75 μM and saturated with 1% BSA. HUVECs were seeded at a density of 2 × 10^4^ cells per well. The adhered cells were measured by cck-8 kit. The number of HUVECs bound to PB-TUP-P2-coated wells showed a very significant difference from that of P2-coated wells. Fusion with PB-TUP did not affect the interaction between P2 and HUVECs.

Previous research showed that P2 could interact with integrin α5β1 and mediate HUVEC adhesion^23^. We performed the cell adhesion assay with fused peptide PB-TUP-P2 to investigate as to whether this activity of P2 is affected by fusion with PB-TUP. Because of the poor absorption of P2, the adhesion experiment was done with less and gentle wash. As shown in Fig. 9, fusion with PB-TUP did not affect the interaction between P2 and HUVECs. On the contrary, the number of HUVECs bound to PB-TUP-P2-coated wells showed a very significant increase comparing with that of P2-coated wells because of the better absorption to polystyrene surface, which is consistent with Fig. 8. From this experiment, we can see that PB-TUP fused to N-terminus of P2 did not change the confirmation of P2 and did not affect its activity in HUVECs adhesion.

## Discussion

Peptide phage display is a powerful method in discovering specific binding sequences with diverse targets like proteins^28^, enzymes^29^, virus^30^, cells^31^, tissues or organs^32^, nanoparticles^33^, materials^34^. It has wide applications in drug discovering, epitope mapping, vaccine design and so on. However, after several rounds of screening, some peptides fail to bind their targets might also be isolated and those false positive sequence are called target unrelated peptides. The reasons of TUP accumulation are complex and can be categorized as selection related and propagation related. Selection related TUP clones are selected because of their affinity to the components of the screening system such as solid surface, blocking agent or washing buffer^12, 14, 35^. Propagation related TUPs have faster propagation rate and are accumulated by faster growth over the other clones^36–38^. These TUPs may give false positive results of screening and mislead further research work.

In our bio-panning against different targets, a clone with the same peptide sequence was isolated. As there is little chance that the same peptide could bind with three different proteins, we did experiments to verify if it is an unrelated peptide. First, we used different combinations of blocking agents and washing buffers in an ELISA assay. NFM-TBS had the best blocking effect and TBST used as a washing buffer showed the most effective washing result. Different from the other groups, PB-TUP had a high ELISA value under all the conditions, confirming its binding ability to polystyrene surface. Further studies excluded the possibility of binding to nonfat milk or binding of a secondary antibody. It bound with the polystyrene surface in a concentration dependent manner.

This 12-mer peptide was transformed into 7-mer peptide by every 7 continuous amino acid residues forward and backward. All these 7-mer peptide were then submitted to PhD7Faster^39^ (http://immunet.cn/sarotup/cgi-bin/PhD7Faster.pl) program for sequence similarity analysis, with the purpose of finding out if it has a propagation advantage. We also did experiments to compare the propagation rate of this clone with the other clones including the whole library, M13KE phage and another non relevant control clone. Both searching results and experimental studies showed that it is not propagation related TUP. All these studies suggested the accumulation of this clone is due to its specific binding to the polystyrene surface.

Adey first reported that phages that express particular peptides can bind to plastic and be recovered during the elution step. Although any consensus sequence was not identified, the plastic-binding motifs of WXXW^7^, FHXXW^40^ and WXXWXXXW^41^ had been found by different research groups. Furthermore, many other sequences without a clear motif that bind to polystyrene have been reported^36, 41–43^. The sequence of VHWDFRQWWQPS is different from the published plastic-binding motifs although they share a similar structure. It contains three Tyr and rich in other hydrophobic amino acids such as Val, His, Phe and Pro. The side chains of these amino acids form strong hydrophobic interactions with benzene ring structure of polystyrene. We simulated the secondary structure of the peptide by molecular modeling (Fig. 7). This peptide has a stretched structure with aromatic rings of Tyr and Phe as side chains, suggesting that π -π stacking through the phenyl group may play a major role in the binding to polystyrene. Imidazole ring in histidine, pyrrole ring in proline and isopropyl group of valine may also contribute to this interaction. Trp, Phe, Pro, and Val, which exist in this peptide sequence, are the most frequent appeared amino acids in polystyrene binding sequences according to the published papers^7, 44–46^.

This peptide also contains several basic and acidic amino acids such as histidine, arginine and aspartic acid, which are not popular in polystyrene bind sequence. But these amino acids together with the other two glutamines may be ionized and function in the binding with plate by electrostatic adsorption. This was confirmed by the effect of altering pH on polystyrene binding in an ELISA test. When pH became acidic or basic, the binding of PB-TUP was increased, although control groups were not affected. This did not agree with the previous report^47^. The reason should be the enrichment of ionizing amino acids of this peptide. By analyzing the peptide conformations dynamically at different pH, we found the peptide formed different secondary structures. We speculated that the stable structure of α-helix (Fig. 7B) and β-turn (Fig. 7D) formed at acidic and basic pH helped in its binding with polystyrene.

Phage display is powerful method. However, if researchers are not careful, the contamination of target unrelated peptides can mislead the research work and they can come up to false results. For example, TUPs are the main noise for epitope mapping and there are now quite a few programs for mimotope based epitope mapping, but none of them has a procedure to scan, report and exclude target-unrelated peptides^48–51^. SAROTUP (Scanner And Reporter Of Target-Unrelated Peptides, http://immunet.cn/sarotup/index.html) is a free website which is designed for finding, reporting, and precluding possible target-unrelated peptides^52^. By peptide sequence searching, PB-TUP sequence did not match any known TUP motif. Interestingly, by searching MimoDB database of this website (http://immunet.cn/sarotup/cgi-bin/MimoSearch.pl), we found this sequence was identical with three submitted peptide sequences. This peptide sequence was found as an IGF2R binding peptide with high affinity by Cheng’s group^15^ (Mimotope Set ID 2874) and the same sequence was identified by Guo as a mimotope of polyclonal antibody against Deinagkistrodon acutus venom^16^ (Mimotope Set ID 3027). Recently it was reported again in epitope mapping of kappa fractions of polyclonal IgG of a multiple myeloma patient^17^ (Mimotope Set ID 3051). In our laboratory, it appeared in the bio-panning against three different targets. We can see that this sequence can be selected with a high frequency in Ph.D.-12 peptide library screening. It is important to report this sequence to avoid further misleading and it will be beneficial for researchers to distinguish it from the real target binding peptides.

However, those polystyrene binding peptides have advantages in peptide or protein immobilization. Peptides, especially those with a low molecular weight, are rarely used as capture agents in direct ELISA because of their poor absorbance to polystyrene. In peptide detecting or analysis, surface-modified plates can improve peptide absorbance in direct ELISA, but with increased cost^53^. Indirect ELISA is often used in peptide analysis, but in this assay the target peptides have to be covalently linked to BSA as a coating agent^24^. Recently, symmetrical carrier landscape phage has been developed for peptide immobilization and been used successfully in microarray^4^. However pre-selection of landscape library for specific peptide ligands is necessary. Fusion of a polystyrene binding peptide with a target peptide or protein provides a convenient and efficient choice. One polystyrene binding peptide sequence can be fused with different targets for immobilization. The fused peptide PB-TUP-P2 demonstrated a significantly increased binding comparing with the native peptide P2 and PB-TUP-P2 showed a typical concentration dependent binding curve. BSA-P2 also displayed a good absorption to polystyrene surface, but different dilutions of BSA-P2 did not show difference in ELISA value in our experiment. And the molar ratio of BSA and P2 are not certain. Proteins adsorbed on a solid surface such as polystyrene sometimes lose their activities because of conformational change and disordered orientation, which render the functional sites disrupted or inaccessible^54^. Fusion target proteins with polystyrene binding peptide can solve this problem and provides an easy way for site specific or well-oriented immobilization onto the PS plates while maintaining high biological activity. In our study, addition of PB-TUB to the N-terminus of P2 did not affect its functional site in HUVEC adhesion and the increased binding to polystyrene even enhanced this activity.

In summary, we found incidentally a polystyrene binding peptide sequence (PB-TUP) in phage display screening. The polystyrene binding property of PB-TUP was confirmed and other possibilities were excluded. By fusion to the N-terminus of a previously studied peptide P2, PB-TUP showed its potential in peptide immobilization.

## Materials and Methods

### Library screening

Target proteins (folate receptor-α, programmed death 1 and programmed death-ligand 1) were dissolved in carbonate buffer pH 9.6 and coated on polystyrene 96-well microtiter plates (Corning, Inc., NY, USA) overnight at 4 °C at a concentration of 50–150 μg/mL. The next day, each well was blocked with 250 μL 3% (w/v) non-fat milk (NFM) (Sangon Biotech Co., Ltd., Shanghai) for 2 hours at 37 °C. After washing 5 times with TBS containing 0.05% (v/v) Tween 20 (TBST), 100 μL Ph.D.-12 peptide library (New England BioLabs, Inc., USA) aliquot containing 10^11^ phages was added to each well and the plate was incubated for 1 hour at 37 °C. After washing 10 times with TBST and 3 times with TBS to remove the unbounded phages, the target-bound phages were eluted with 150 μL 100 mM Glycine-HCl (pH 2.2) and neutralized immediately with 9 μL 2 M Tris-HCl (pH 9.1). 5 μL of eluate was used for titering and the rest was used to infect E. coli ER2738 host strain (New England BioLabs, Inc., USA) for amplification.

### Phage titering and amplification

150 μL Phage suspension was added to 25 mL ER2738 cell culture in log phase (OD600 0.4-0.6). After incubation with vigorous shaking for 4.5–5 hours at 37 °C, the culture was centrifuged at 10,000 rpm for 10 minutes at 4 °C. The supernatant was participated with 1/5 volume 20% (w/v) PEG-8000/2.5 M NaCl overnight at 4 °C. On the next day, participated phages were collected by centrifugation at 12,000 rpm for 15 minutes at 4 °C and resuspended in 1% (w/v) bovine serum albumin (BSA) (Sangon Biotech Co., Ltd., Shanghai) for titering and the next round bio-panning. For titering, 10 μL serial dilutions of phage solutions were added into 200 μL log phase ER2738 cell cultures. After incubation for 5–10 minutes, the culture was mixed with 3 mL 45 °C top agar and poured onto pre-warmed LB/IPTG/X-gal plate immediately. After incubation of the plates overnight at 37 °C, blue plaques were counted and phage titer as plaque forming units (pfu) was calculated.

### Phage ELISA assay

96-well polystyrene microtiter plates were coated with a solution of target protein (5 μg/mL) in 0.05 M sodium bicarbonate (pH 9.6) and the plate was kept overnight at 4 °C. After washing the plate three times, the plate was blocked with 0.1 M PBS (pH 7.4) containing 3% NFM at 37 °C for 2 hours (200 μL/well). Individual phage clones were amplified and 10^10^ phages were added to each well (100 μL/well) and incubated for 2 hours at 37 °C. After washing the plate for 3 times with TBST and 3 times with TBS, 100 μL of HRP-conjugated anti-M13 antibody (1:5000, Sino Biological, Inc., Beijing) was added and the plates were incubated for 2 hours at 37 °C. After washing three times, the tetramethylbenzidine (TMB) (Sangon Biotech Co., Ltd., Shanghai) substrate (100 μL/well) was added and the reaction lasted for 15 minutes. The reactions were stopped with 2 M sulfuric acid (50 μL/well). The absorbance of each well at 450 nm was detected with an automated ELISA reader. Phage clones with high absorbance were selected for sequencing.

### Polystyrene binding study

96-well polystyrene microtiter plates were blocked at 37 °C for 2 hours with different blocking buffers including PBS, 0.05% PBST, TBS, 0.05% TBST, 3% BSA-TBS and 3% NFM-TBS separately. 10^10^ pfu of possible polystyrene binding clone PB-TUP, Ph.D.-12 peptide library, M13KE (LacZa(-) wild-type M13 phage, New England BioLabs, Inc., USA), and a non-relevant phage D12 clone was added into the microtiter plates as a control group and the plates were incubated overnight at 4 °C. Then the plates were washed 5 times with the same buffer which was used for incubation, and the relative number of bound phage was determined by phage ELISA.

### Evaluation of phage binding to blocking buffer

96-well polystyrene microtiter plates were blocked at 37 °C for 2 hours with different concentration (0.00, 0.01, 0.10, 0.20, 0.40, 0.80, 1.60, 3.20%) (w/v) NFM-TBS. 10^10^ pfu of PB-TUP, Ph.D.-12 peptide library, M13KE, and D12 clone were added to the microtiter plates and incubated overnight at 4 °C. Next day, the plates were washed 3 times with TBST and 3 times with TBS and the relative number of bound phage was determined by phage ELISA.

### Evaluation of antibody binding to polystyrene

In order to avoid the false signal caused by antibody binding to polystyrene, we used extra 3% NFM-TBS blocking for 1 hour before adding HRP-conjugated anti-M13 antibody in the ELISA procedure and less stringent washing was used in ELISA. Furthermore, after phage clones were added into blocked wells and incubated for 2 hours, phages were eluted with 150 μL 100 mM Glycine-HCl (pH 2.2) for 10 minutes and neutralized with 9 μL 2 M Tris-HCl immediately. Phage titer was determined and compared with the absorbance of the ELISA assay.

### Propagation rate of phage clone

To compare the phage propagation rates, 2 mL overnight culture of ER2738 was diluted 1:100 in LB medium. 10^6^ PB-TUP, Ph.D.-12 peptide library, M13KE, and D12 clone were added separately into the logarithmic growth phase culture for infection and amplification. The cultures were shaken vigorously for 5 hours at 37 °C. During the amplification, 50 μL samples were taken from the culture every 1 hour for titration.

### Concentration dependent binding to polystyrene

Different concentrations of PB-TUP, Ph.D.-12 library, M13KE, and D12 clone were individually added into the blocked microtiter wells. ELISA was used to determine the phage binding and the titer of the recovered phage was determined by LB/IPTG/X-gal plate.

### Effect of pH changes on phage binding

In order to study the effect of pH on phage binding to polystyrene, 10^10^ pfu of PB-TUP, Ph.D. -12 peptide library, M13KE, and D12 clone were incubated in solutions with varying pH values and were added to the blocked wells of the polystyrene microtiter plate. Phage binding was tested by ELISA.

### Molecular modelling

The initial structure of the peptide was built using ChemDraw, and then stimulated by molecular dynamics (MD) using Gromacs 5.0.2 at different pH. Root-mean-square-deviation (RMSD) plot was used to evaluate the time needed for this system to reach equilibrium.

### Immobilization of peptide P2 onto a polystyrene plate through fusion with PB-TUP

Peptide P2 and PB-TUP-P2 was synthesized by GL Biochem (Shanghai, China) with a purity of over 95%. Peptide sequence of P2 and the appended sequence with an N-terminal PB-TUP sequence were listed in [Table 1]. Peptide P2 was conjugated with BSA by glutaraldehyde coupling. Specifically, 50 µl glutaraldehyde (25%) was diluted to 5 ml with deionized water. 120 µl diluted glutaraldehyde was added to a mixture of 0.5 ml peptide P2 (4 mg/ml) and 0.5 ml BSA (2 mg/ml). The mixture was kept in dark and rotated gently for 4 h. Then 16 µl glycine solution (1 M) was added and the BSA-P2 was ready for use after 1 h rotation. P2 polyclonal antibody was prepared as previously described^24^. Maxisorp polystyrene 96-well plate was coated with different concentrations of P2 or PB-TUP-P2 (5 μM–100 μM) or different dilutions of BSA-P2 (100–5000) in 0.05 M sodium bicarbonate (pH 9.6) and the plate was kept overnight at 4 °C. After washing the plate three times, the plate was blocked with TBS containing 3% NFM at 37 °C for 1.5 h. P2 specific antibody (1:1000) was added into the wells and the plate was incubated at 37 °C for 1.5 h. After washing the plate three times, 100 μL horseradish peroxidase-conjugated goat anti-rabbit IgG (1:2000) was added and the plate was incubated for 1 h at 37 °C. After washing three times, the tetramethylbenzidine (TMB) (Sangon Biotech Co., Ltd., Shanghai) substrate (100 μL/well) was added and the reaction lasted for 15 minutes. The reactions were stopped with 2 M sulfuric acid (50 μL/well). The absorbance of each well at 450 nm was detected with an automated ELISA reader.

### HUVEC adhesion

Polystyrene 96-well cell culture plates were coated with P2 or PB-TUP-P2 at a concentration of 75 μM at 4 °C overnight. The remaining adhesion sites were saturated with 1% BSA at 37 °C for 2 h. HUVECs (Human Umbilical Vein Endothelial Cells) were cultured to 80% confluence and starved overnight in serum-free medium. Next day, the cells were harvested and seeded in each well at a density of 2 × 10^5^ cells/mL, 100 μL per well. After the plate was maintained at 37 °C with 5% CO_2_ for 1.5 h, the cell medium was discarded carefully and unattached cells were removed by gentle washing with PBS. The number of adhered cells was measured by cck-8 kit. 100 μL PBS with 10 μL cck-8 kit solution was added to each well and the plate was incubated for 4 h at 37 °C. The absorbance of each well at 450 nm was detected with an automated ELISA reader. For each condition, there were five repetitive wells.

### Statistical analysis

All statistical analyses were performed by using GraphPad Prism 6.0 software (GraphPad software Inc., San Diego, CA, USA). Unpaired two-tailed between student’s t-test was used to analyze the differences between means. All data are expressed as the mean ± S.D. The error bars represent the standard deviation of three independent determinations. P < 0.05 is considered statistically significant. *P < 0.05, **P < 0.01, ***P < 0.001.
